# Two-Dimensional Dynamic Logic Resource Allocation for Scalable RIS Channel Emulation

**DOI:** 10.3390/s26030813

**Published:** 2026-01-26

**Authors:** Dan Fei, Haobo Zhang, Chen Chen, Hao Zhou, Peng Zheng, Guoyu Wang, Cheng Li, Jiayi Zhang, Zhaohui Song, Bo Ai

**Affiliations:** 1 State Key Laboratory of Advanced Rail Autonomous Operation, Beijing Jiaotong University, Beijing 100044, China; dfei@bjtu.edu.cn; 2 Nanjing Rongcai Transportation Technology Research Institute Co., Ltd., Nanjing 210012, China; 3 School of Electronic and Information Engineering, Beijing Jiaotong University, Beijing 100044, China; 25115055@bjtu.edu.cn (H.Z.);; 4 Space Information Research Institute and Zhejiang Key Laboratory of Space Information Sensing and Transmission, Hangzhou Dianzi University, Hangzhou 310005, China

**Keywords:** channel emulation, dynamic resource allocation, scalability, queueing theory, game theory, hardware-in-the-loop (HIL), resource pooling

## Abstract

This paper addresses the critical scalability challenge in Hardware-in-the-Loop (HIL) channel emulation for massive RIS-assisted 6G environments. We propose a Two-Dimensional Dynamic Logic Resource Allocation (2D-DLRA) architecture that decouples physical RF ports from baseband processing resources through hierarchical pooling at both the session level and the multipath level. By jointly virtualizing Logical Units (LUs) and Multipath Processing Units (MPUs), the proposed architecture overcomes the dual inefficiency of port underutilization and path-level sparsity inherent in conventional static designs. A rigorous analytical framework combining hierarchical queuing theory and non-cooperative game theory is developed to characterize system capacity, blocking probability, and user contention under heterogeneous workloads. Simulation results demonstrate that, under a strict QoS constraint of 1% blocking probability, the proposed 2D-DLRA architecture achieves a multi-fold increase in supported user capacity compared to static allocation with the same hardware resources. Moreover, for an end-to-end emulation error threshold of 3%, 91.8% of users meet the QoS requirement, compared to only 73.6% in static architectures. The results further show that dynamic pooling enables near-saturated hardware utilization, in contrast to the single-digit utilization typical of static designs in sparse RIS scenarios. These findings confirm that 2D-DLRA provides a scalable and hardware-efficient solution for large-scale RIS channel emulation, offering practical design guidelines for next-generation 6G HIL testing platforms.

## 1. Introduction

The evolution towards sixth-generation (6G) wireless networks is driving a paradigm shift from merely adapting to the wireless channel to actively controlling it, materialized by the deployment of Reconfigurable Intelligent Surfaces (RISs) [1,2,3,4,5,6,7,8,9,10]. By leveraging massive arrays comprising hundreds or even thousands of low-cost reflecting elements, RISs empower the creation of Smart Radio Environments (SREs) with programmable signal propagation [11,12]. However, this technological leap poses an unprecedented challenge for hardware-in-the-loop (HIL) channel emulation, as the required emulation scale increases dramatically [13,14]. Validating RIS-assisted systems requires emulating channels with a massive number of spatial nodes, yet the physical propagation environment often exhibits sparsity in the angular and delay domains. This unique characteristic—massive ports but sparse effective paths—renders the architectural scalability of channel emulators the primary bottleneck in the design and optimization of next-generation wireless technologies [15,16,17,18,19,20].

**Figure 1:**
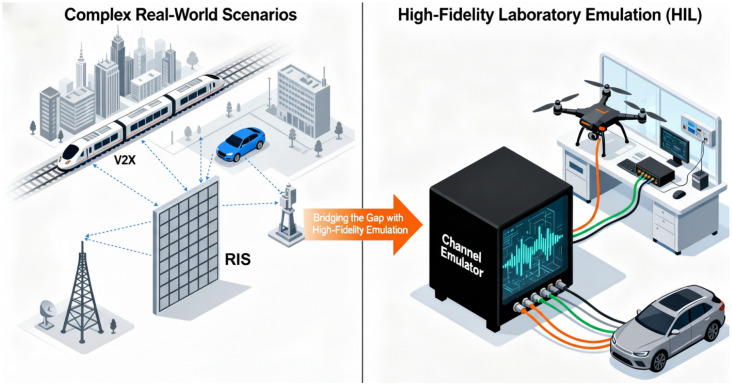
Wireless channel emulation system reproduces the real environment with high fidelity.

However, conventional emulator architectures are predicated on a paradigm of static resource allocation, which leads to a profound, two-dimensional inefficiency when applied to RIS scenarios [21,22,23]. The first dimension arises from the rigid, one-to-one mapping of physical RF ports to dedicated processing chains (Logical Units, LUs) [24], causing costly FPGA resources to be “stranded” in scenarios with sporadic user activity [25,26,27]. As illustrated in Figure 1, given that an RIS may contain thousands of elements, statically allocating a dedicated, power-hungry FPGA chain to each element is prohibitively expensive and physically infeasible. A second, more subtle dimension of inefficiency exists within each LU, where a fixed, worst-case number of Multipath Processing Units (MPUs) is allocated [28], regardless of the variable path requirements of different channel models [29]. However, RIS-assisted channels typically exhibit high sparsity, where each element contributes only to a limited number of propagation paths. This compounding inefficiency, where over 95% of computational resources can be idle, renders traditional architectures economically infeasible for future massive connectivity scenarios [28]. To address this critical scalability challenge, and inspired by theories of socially optimal resource allocation and queue management [30,31], this paper proposes a novel Two-Dimensional Dynamic Logic Resource Allocation (2D-DLRA) architecture tailored for massive and sparse connectivity scenarios like RISs. Parallel to advancements in hardware emulation, the theoretical analysis of these complex systems, particularly for resource management and Quality of Service (QoS) assurance, has become critical [32,33]. The use of queueing theory, in particular, has become a widespread and powerful tool for modeling traffic, optimizing data transfer, and analyzing performance in diverse network environments such as IoT and mobile cloud computing [34,35,36].

While prior works have explored dynamic allocation [37], our approach introduces a hierarchical, resource-pooled paradigm that fundamentally redesigns the allocation mechanism at two distinct granularities. This architecture abandons rigid static mapping to perform on-demand, fine-grained allocation of both session-level Logical Units (LUs) and path-level Multipath Processing Units (MPUs), thereby simultaneously resolving both dimensions of the inefficiency problem. The core contributions of this work are multifaceted and summarized as follows:

- We propose a novel 2D-DLRA architecture that overcomes the two-dimensional inefficiency of static designs through a hierarchical, resource-pooled paradigm, enabling scalability of the channel emulator [38,39].
- We develop a rigorous mathematical model for the 2D-DLRA architecture based on hierarchical queueing theory. This framework yields a rich set of analytical formulas for key performance indicators (KPIs), including blocking probability, resource utilization, and system capacity.
- We introduce a non-cooperative game-theoretic model to analyze the micro-behavioral dynamics of resource contention among heterogeneous users or RIS subarrays, providing deep insights into the system’s Nash Equilibrium and fairness.

The remainder of this paper is organized as follows. Section 2 details the 2D-DLRA architecture and its operational mechanism. Section 3 establishes the performance modeling framework based on hierarchical queueing theory. Section 4 introduces the game-theoretic analysis of resource contention. Section 5 presents the comprehensive joint analysis and experimental validation results. Finally, Section 6 concludes the paper.

## 2. The 2D-DLRA Architecture and Operational Mechanism

This chapter establishes the foundational concepts of our proposed architecture. We begin by providing a deep and formal analysis of the fundamental scalability challenges inherent in traditional emulator designs, precisely defining the two-dimensional nature of their inefficiency. This analysis serves as the primary motivation for the paradigm shift towards the hierarchical, resource-pooled architecture that will be detailed subsequently.

### 2.1. The Scalability Challenge and the Static Allocation Bottleneck

#### 2.1.1. The First Dimension of Inefficiency: Stranded Session-Level Resources

The conventional emulator architecture is characterized by a rigid, one-to-one mapping between its *N* physical Radio Frequency (RF) ports and an equal number of dedicated, independent baseband processing chains, which we term Logical Units (LUs). While simple to implement, this static design creates a fundamental bottleneck we refer to as the stranded session-level resource problem.

Let the state of the *i*-th RF port be a binary random variable $S_{i}\in \left\{0,1\right\}$, where $S_{i}=1$ if the port is active (i.e., a signal is present) and $S_{i}=0$ if it is idle. The activity factor, or the probability of the port being active, is $p_{\mathrm{act}}=P\left(S_{i}=1\right)$. The total hardware cost of the emulation subsystem, $H_{\mathrm{total}}$, is directly proportional to the number of physical ports, *N*, as each port requires a dedicated LU:

(1) $$H_{\mathrm{static}}\propto N$$

The utilization of the *i*-th LU, $\eta_{i}$, is equivalent to the activity factor of its corresponding port, $\eta_{i}=p_{\mathrm{act}}$. The overall system resource utilization, $\eta_{\mathrm{system}}^{\mathrm{static}}$, is the average utilization across all LUs. Assuming statistically identical and independent user behavior, the expected number of active links is $E\left[\sum S_{i}\right]=N\cdot p_{\mathrm{act}}$. The overall resource utilization is therefore:

(2) $$\eta_{\mathrm{system}}^{\mathrm{static}}=\frac{N_{\mathrm{act}}}{N}=p_{\mathrm{act}}$$

where $N_{\mathrm{act}}$ is the number of active LUs and *N* is the total number of LUs.

This simple but powerful result reveals the core inefficiency: the system’s overall hardware utilization is capped by the activity factor of a single user. In typical wireless scenarios like V2X or massive IoT, the activity factor is very low (e.g., $p_{\mathrm{act}}\ll 0.1$). This implies that for over 90% of the time, the expensive and power-hungry FPGA resources dedicated to each port are stranded—powered on but performing no useful computation. This directly limits the achievable port density for a given hardware budget and makes scaling to scenarios with thousands of sporadic links prohibitively expensive. This constitutes the first, and most commonly understood, dimension of the static allocation bottleneck.

#### 2.1.2. The Second Dimension of Inefficiency: Stranded Path-Level Resources

A more subtle, yet equally critical, layer of inefficiency exists within each of the statically allocated LUs. An LU is primarily composed of a set of digital signal processing resources responsible for implementing the fading process for each multipath component of a channel model. We term these resources Multipath Processing Units (MPUs). A traditional LU is designed with a fixed, maximum number of MPUs, *P*, sufficient to handle a worst-case, highly complex channel model (e.g., *P* = 24 for a standardized 3GPP TDL model).

This fixed internal allocation creates the second dimension of resource stranding. The actual number of multipaths required by a channel model is not a constant; it is a random variable that depends on the propagation environment. Let the number of paths required by the channel model for the *i*-th link be the random variable $p_{i}$, where its probability mass function is $q_{p}=P\left(p_{i}=p\right)$ for p∈{1,2,…,P}. For example, a Line-of-Sight (LoS) channel might only require $p_{i}=2$ paths, while a rich Non-Line-of-Sight (NLOS) channel might require $p_{i}=20$ paths.

In a static architecture, even if a link *i* is active ($S_{i}=1$), the utilization of the MPU resources within its dedicated LU is not 100%. The internal MPU utilization for an active link *i*, $\eta_{\mathrm{MPU},i}$, is given by the ratio of required paths to available paths:

(3) $$\eta_{\mathrm{MPU},i}=\frac{p_{i}}{P}$$

The overall system-wide MPU utilization, $\eta_{\mathrm{MPU}}^{\mathrm{static}}$, is the expectation of this value, averaged over all LUs and all states (active/idle). Since the link state $S_{i}$ and the path requirement $p_{i}$ are independent random variables:

(4) $$\eta_{\mathrm{MPU}}^{\mathrm{static}}=E\left[\frac{S_{i}p_{i}}{P}\right]=\frac{E\left[S_{i}\right]\cdot E\left[p_{i}\right]}{P}=p_{\mathrm{act}}\cdot \frac{\bar{p}}{P}$$

where $\bar{p}=E\left[p_{i}\right]=\sum_{p=1}^{P}p\cdot q_{p}$ is the average number of multipaths required per channel model.

Equation (4) exposes the compounding inefficiency. The total utilization of the fine-grained computational resources is the product of two factors, both typically much less than 1: the user activity factor ($p_{\mathrm{act}}$) and the average multipath requirement ratio ($\bar{p}/P$). For a scenario with $p_{\mathrm{act}}=0.1$ and an average path requirement of $\bar{p}=8$ out of a maximum of P=24, the total MPU utilization would be a mere $\eta_{\mathrm{MPU}}^{\mathrm{static}}=0.1\times \left(8/24\right)\approx 3.3\%$. This means that over 96% of the silicon resources are effectively wasted.

This two-dimensional inefficiency provides the clear and compelling motivation for a paradigm shift. A truly scalable and efficient architecture must not only share resources between users (addressing the first dimension) but must also allow for the fine-grained, on-demand allocation of computational resources based on the specific, variable demands of the channel models being emulated (addressing the second dimension). This is the foundational principle of our 2D-DLRA architecture.

### 2.2. The 2D-DLRA Architecture: A Paradigm Shift to Hierarchical Pooling

To overcome the multi-dimensional inefficiencies of static allocation, we propose a fundamental paradigm shift in emulator architecture. The Two-Dimensional Dynamic Logic Resource Allocation (2D-DLRA) design moves away from rigid, dedicated hardware chains to a flexible, software-defined model based on the principles of decoupling and hierarchical resource virtualization. This approach allows for the on-demand, fine-grained allocation of computational resources at two distinct granularities, thereby maximizing hardware utilization and enabling unprecedented scalability [32,33,40].

**Figure 2:**
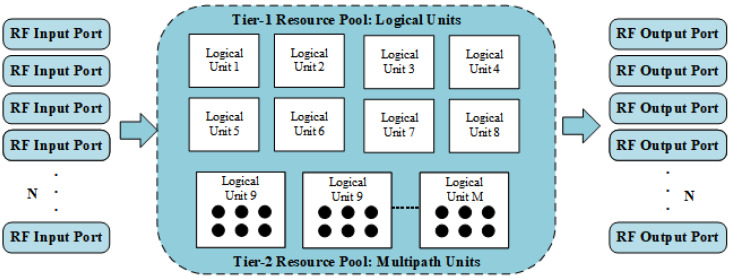
Architecture of the Two-Dimensional Dynamic Logic Resource Allocation (2D-DLRA) System.

The architecture is formally defined by its two-tiered pooling structure, as illustrated in Figure 2.

#### 2.2.1. The Principle of Decoupling and Virtualization

The core philosophy of 2D-DLRA is the complete separation of the physical layer (the *N* RF ports) from the computational layer (the processing resources). We formally define the set of physical ports as N={1,2,…,N} and the set of available Logical Units as M={1,2,…,M}.

In a static architecture, a fixed, bijective mapping function $\Phi_{\mathrm{static}}:N\to M$ (where N=M) exists. In our 2D-DLRA paradigm, this mapping is dynamic and time-varying. We introduce a time-dependent mapping function $\Phi_{L}\left(t\right)$ that assigns a subset of active ports, $N_{\mathrm{active}}\left(t\right)\subseteq N$, to a subset of available logical units.

(5) $$\Phi_{i}\left(t\right):N_{\mathrm{active}}\left(t\right)\to M_{\mathrm{assigned}}\left(t\right)$$

where $M_{\mathrm{assigned}}\left(t\right)\subseteq M$ and $|N_{\mathrm{active}}\left(t\right)|=|M_{\mathrm{assigned}}\left(t\right)|\leq M$.

This decoupling enables *virtualization*: the *N* physical ports are presented to the user, while the *M* underlying logical units are abstracted into a shared, fungible pool.

#### 2.2.2. Tier-1 Resource Pool: The Logical Unit (LU) Pool

The first tier of our architecture is the Logical Unit (LU) pool, which consists of *M* independent, reconfigurable processing engines. The primary purpose of this tier is to serve incoming, active communication links on a one-to-one basis. When a signal appears at an RF port, the system allocates one entire LU from this pool to service the link for its entire duration.

This pooling strategy directly addresses the stranded session-level resource problem described in Section 2.1.1. We can define a Port-to-Resource Ratio (PRR) as a measure of the system’s multiplexing capability:

(6) $$PRR=\frac{N}{M}$$

A PRR greater than 1 indicates that the system is leveraging statistical multiplexing. For example, a system with N=32 and M=16 has a PRR of 2, implying it can support twice as many physical interfaces as it has processing chains, under the assumption that not all interfaces are active simultaneously. The performance of this tier—specifically, the probability that an incoming request finds no available LU ($P_{B1}$)—is governed by the total offered traffic load, a relationship that will be formally modeled in Section 3.

#### 2.2.3. Tier-2 Resource Pool: The Multipath Processing Unit (MPU) Pool

The second tier of our architecture addresses the more subtle, internal dimension of inefficiency. Each of the *M* Logical Units contains a set of *P* reconfigurable Multipath Processing Units (MPUs), which are the fine-grained computational elements (e.g., complex multipliers, adders, interpolators) responsible for realizing individual fading paths.

Instead of treating these MPUs as captive resources within each LU, we introduce the concept of a system-wide virtualized MPU pool. Let the set of MPUs within the *j*-th LU be denoted by $P_{j}$, where $|P_{j}|=P$. The virtualized system pool, C, is the union of all MPU resources across all LUs.

(7) $$C=\overset{M}{\underset{j=1}{⋃}}P_{j}$$

The total capacity of this virtualized pool is the total number of MPUs in the system:

(8) C=|C|=M×P

When a request from link *i* is assigned to an LU, the 2D-DLRA architecture does not statically allocate all *P* internal MPUs to it. Instead, based on the channel model’s specific requirement of $p_{i}$ paths, the system performs a second-tier dynamic allocation. It assigns a subset of MPUs, $C_{\mathrm{assigned},i}\subseteq C$, of size $p_{i}$ to the task.

(9) $$|C_{\mathrm{assigned},i}|=p_{i}\leq P$$

This two-tiered, hierarchical pooling strategy ensures that resources are allocated on demand at both a coarse-grained (LU) and a fine-grained (MPU) level. This combats both dimensions of static allocation inefficiency, paving the way for a truly resource-aware and scalable channel emulation architecture. The performance of this second-tier allocation will be rigorously analyzed in Section 3.

### 2.3. Operational Mechanism of the 2D-DLRA System

The 2D-DLRA architecture is realized through a real-time, event-driven operational mechanism orchestrated by a Central Resource Manager (CRM). This mechanism translates the architectural principles of hierarchical pooling into a dynamic, state-aware workflow that handles the complete lifecycle of a service request, from initial signal detection to final resource de-allocation. The process can be formally described in three distinct stages.

#### 2.3.1. Stage 1: Signal Detection and Requirement Characterization

The operational workflow is initiated by the arrival of a signal at one of the *N* physical RF ports. Each port, i∈N, is continuously monitored by a lightweight signal detection module.

- **Signal Detection**: The detection process is modeled as a hypothesis test to distinguish a valid signal from background noise. Let $x_{i}\left(t\right)$ be the digitized signal at the input of port *i*. The system tests:
  - $H_{0}$: Signal absent (only noise is present).
  - $H_{1}$: Signal present.
  A common method to decide between these hypotheses is an energy detector, where the decision statistic, $E_{i}$, is compared against a predefined threshold, γ.
  (10) $$E_{i}=\frac{1}{T_{\mathrm{obs}}}\int_{t-T_{\mathrm{obs}}}^{t}{\left|x_{i}\left(\tau \right)\right|}^{2}d\tau$$
  If $E_{i}>\gamma$, the system declares the presence of a signal, triggering the characterization stage.
- **Requirement Characterization**: Upon detection of a signal, the system must characterize its resource requirements in the two dimensions of our architecture. Let the service request associated with the signal at port *i* at time *t* be denoted by $R_{i}\left(t\right)$. The characterization engine populates the request with the following parameters:
  - **Tier-1 Requirement** ($r_{LU}$): The requirement for a Logical Unit is binary and implicit. The very existence of a valid request $R_{i}\left(t\right)$ implies the need for exactly one LU. We can denote this requirement as $r_{LU}\left(R_{i}\right)=1$.
  - **Tier-2 Requirement** ($r_{MPU}$): The requirement for Multipath Processing Units is more complex and depends on the specific channel model to be emulated for this link. Let the set of all available channel models be CM. The user pre-configures a mapping $\Psi :i\to {\mathrm{CM}}_{k}$ that associates port *i* with a specific channel model ${\mathrm{CM}}_{k}$. Each model has an intrinsic complexity, defined by the number of multipath components it contains. We define a function Ω:CM→{1,2,…,P} that returns the number of required MPUs for any given model. The Tier-2 requirement, $p_{i}$, is therefore determined deterministically.

(11) $$p_{i}=r_{\mathrm{MPU}}\left(R_{i}\right)=\Omega \left(\Psi \left(i\right)\right)$$

The fully characterized request, $R_{i}\left(t\right)=\left\{r_{LU},r_{MPU}\right\}$, is then forwarded to the Central Resource Manager.

#### 2.3.2. Stage 2: Hierarchical Resource Allocation by the Central Manager

The Central Resource Manager (CRM) is the core decision-making entity of the 2D-DLRA system. It maintains the real-time state of both resource pools. Let the set of occupied LUs at time *t* be $M_{\mathrm{occ}}\left(t\right)$ and the set of occupied MPUs be $C_{\mathrm{occ}}\left(t\right)$. The state of the system is the tuple $S\left(t\right)=\left[|M_{\mathrm{occ}}\left(t\right)|,|C_{\mathrm{occ}}\left(t\right)|\right]$. Upon receiving the request $R_{i}\left(t\right)$, the CRM executes a two-stage allocation logic.

- **Tier-1 Allocation Attempt (LU Allocation)**: The CRM first checks the availability of resources in the Tier-1 pool. A request is admitted at this stage if and only if the number of currently occupied LUs is less than the total number of LUs, *M*.
  Admit at Tier-1 if:
  (12) $$|M_{\mathrm{occ}}\left(t\right)|<M$$
  If this condition is not met, the request $R_{i}\left(t\right)$ is blocked (in a loss system) or placed in a queue (in a waiting system). This corresponds to a Tier-1 Blocking Event.
- **Tier-2 Allocation Attempt (MPU Allocation)**: If the request is admitted at Tier-1, an LU (say, $LU_{j}$) is tentatively assigned to it. The CRM then proceeds to the second stage, checking for resource availability in the system-wide virtualized MPU pool. Admission at this stage requires that the number of available MPUs is sufficient to meet the request’s demand, $p_{i}$.
  (13) $$|C_{\mathrm{occ}}\left(t\right)|+p_{i}\leq C$$
  If this condition is met, the allocation is confirmed, and the system state is updated: $|M_{\mathrm{occ}}\left(t\right)|\to |M_{\mathrm{occ}}\left(t\right)|+1$ and $|C_{\mathrm{occ}}\left(t\right)|\to |C_{\mathrm{occ}}\left(t\right)|+p_{i}$. If the condition is not met, the request is blocked. This corresponds to a Tier-2 Blocking Event, and the tentatively assigned LU is immediately released back to the pool.

#### 2.3.3. Stage 3: Dynamic Link Formation and Resource De-Allocation

- **Dynamic Link Formation**: Once a request is successfully admitted at both tiers, the CRM instructs the underlying FPGA hardware to form the physical data path. A high-speed, reconfigurable cross-connect within the FPGA fabric is configured to route the digitized data stream from the physical input port *i* to the newly assigned Logical Unit, $LU_{j}$. Simultaneously, the internal resources of $LU_{j}$ are configured to instantiate exactly $p_{i}$ active Multipath Processing Units, while the remaining $P-p_{i}$ MPUs within that LU remain dormant and conceptually available to the system-wide pool.
- **Resource De-allocation**: The CRM continuously monitors the status of the active signal at port *i*. When the signal terminates (i.e., the energy statistic $E_{i}$ falls below the threshold γ for a specified duration), a de-allocation procedure is initiated.
  (14) $$\begin{array}{cc} |M_{\mathrm{occ}}\left(t\right)| & \to \;|M_{\mathrm{occ}}\left(t\right)|-1,\\ |C_{\mathrm{occ}}\left(t\right)| & \to \;|C_{\mathrm{occ}}\left(t\right)|-p_{i}. \end{array}$$

The FPGA cross-connect is reconfigured to tear down the data path, and both the LU and its associated MPUs are gracefully released back into their respective shared pools, becoming immediately available for subsequent service requests. This completes the lifecycle and ensures the “on-demand” nature of the resource allocation.

Algorithm 1 summarizes the logical operation flow of the CRM rather than a hardware scheduling or optimization algorithm.

**Algorithm 1** CRM Operation Procedure in the 2D-DLRA Architecture**Input:** Available port resources $R_{p}$, available multipath resources $R_{m}$, monitoring period *T* **Output:** Dynamic mapping between active paths and emulation resources  1: **Initialization:**
  2:  Initialize port-level resource pool $R_{p}$
  3:  Initialize multipath-level resource pool $R_{m}$
  4:  Initialize active path set P=∅  5:  Initialize resource occupancy state and mapping table            System is running   6:   Wait for the next monitoring period *T*  7:  **Path Arrival Handling:** newly arrived paths $p\in P_{\mathrm{new}}$ available resources exist in $R_{p}$ and $R_{m}$
  8:  Allocate one port resource and one multipath resource to *p*  9:  Update resource occupancy state 10:  Add *p* to active path set P 11:  Block or queue path *p* according to system policy 12:  **Path Departure Handling:** departed paths $p\in P_{\mathrm{dep}}$ 13:  Release the port and multipath resources occupied by *p* 14:  Update resource occupancy state 15:  Remove *p* from active path set P 16:  **State Update:** 17:  Update system statistics for analytical modeling


It should be noted that Algorithm 1 specifies the operational logic of the CRM rather than a scheduling or optimization algorithm.

## 3. Performance Modeling via Hierarchical Queueing Theory

Having established the architecture and operational mechanism of the 2D-DLRA system, we now develop a rigorous mathematical framework to analyze its performance. This chapter employs principles from hierarchical queueing theory to derive analytical expressions for a comprehensive set of Key Performance Indicators (KPIs). These KPIs will quantify the system’s efficiency, capacity, and Quality of Service (QoS), providing powerful predictive tools for system design and planning.

### 3.1. System Model and Formal Definitions

We model the 2D-DLRA system as a hierarchical resource allocation system where service requests arrive and compete for two distinct tiers of resources. The arrival of service requests is modeled as a Poisson process, a widely accepted model for scenarios involving a large number of independent, uncoordinated users.

#### 3.1.1. Formal System and Resource Definitions

The physical and logical resources of the architecture are formally defined as:

- The set of physical RF ports: N={1,2,…,N}, with cardinality |N|=N.
- The Tier-1 pool of Logical Units (LUs): M={1,2,…,M}, with cardinality |M|=M.
- The maximum number of Multipath Processing Units (MPUs) per LU: *P*.
- The total capacity of the virtualized Tier-2 MPU pool: C=M×P.

#### 3.1.2. Traffic and Workload Model

- **Arrival Process**: Service requests arrive at the system according to a Poisson process with a mean aggregate arrival rate of λ (requests per unit time). The total offered traffic load to the system, *A*, measured in Erlangs, is given by:
  (15) A=λ·E[S]
  where E[S]=1/μ is the mean service time of a request.
- **Workload Model (Multipath Demand Distribution)**:
  In this work, the multipath number is modeled as a random counting variable representing the number of simultaneously active and resolvable paths within a channel snapshot. From a system-level perspective, this quantity characterizes the instantaneous computational workload rather than the physical propagation mechanism.
  Among commonly used discrete distributions, the Poisson distribution is particularly suitable for this purpose, as it models the number of independent and rare events occurring within a fixed observation window and requires only a single parameter. Alternative distributions such as binomial or negative binomial would require additional assumptions regarding the total number of potential paths or over-dispersion, which are difficult to justify at the architectural level.
  Moreover, the Poisson distribution naturally arises as the limiting case of the sum of a large number of independent Bernoulli trials with small activation probabilities, which aligns with the sparse nature of effective multipath components. The truncation reflects the finite hardware resources available in practical channel emulators.
  The number of MPUs required by an arriving request is a discrete random variable, *p*, with a probability mass function (PMF) denoted by $\left\{q_{p}\right\}$. This distribution is particularly relevant for RIS-assisted channels, which exhibit high sparsity. While the number of RIS elements is large, the number of significant propagation paths is typically small and time-varying due to beamforming. We model this distribution using a *truncated Poisson distribution*. This reflects the real-world observation that most channel models require a moderate number of paths, with very simple (low *p*) and very complex (high *p*) models being less frequent.

Truncated Poisson PMF for Multipath Demand:

(16) $$q_{p}=\frac{\frac{\lambda_{mp}^{p}e^{-\lambda_{mp}}}{p!}}{\sum_{k=1}^{P}\frac{\lambda_{mp}^{k}e^{-\lambda_{mp}}}{k!}},\;\mathrm{for}\;p\in \left\{1,2,\ldots ,P\right\}$$

Here, $\lambda_{mp}$ is the mean of the underlying (non-truncated) Poisson distribution, representing the average number of paths one might expect in the given emulation scenario (e.g., $\lambda_{mp}=8$ for a typical urban NLOS model). This formula ensures that the probabilities sum to one over the valid range of path requirements, {1,…,P}. The average number of required paths, $\bar{p}$, can be calculated as:

(17) $$\bar{p}=E\left[p\right]=\sum_{p=1}^{P}p\cdot q_{p}$$

This formal model, with its defined system parameters and stochastic workload, provides the complete foundation necessary for the detailed performance analysis of the two resource tiers in the subsequent sections.

### 3.2. Tier-1 Analysis: Logical Unit Blocking Probability

The first tier of the 2D-DLRA system functions as the primary gatekeeper for incoming service requests. It consists of *M* parallel, identical servers (the Logical Units) that serve a total offered traffic load of *A* Erlangs. For our initial and primary analysis, we model this tier as a classic loss system, where a request that arrives when all M servers are occupied is blocked and cleared from the system. This corresponds to the M/G/c/c queue in Kendall’s notation, for which the performance is precisely described by the Erlang *B* formula.

The blocking probability at this tier, denoted as $P_{B1}$, represents the probability that an incoming request finds no available LU and is consequently denied service at the session level. This metric is a fundamental measure of the system’s coarse-grained capacity. It is a function of only two parameters: the total offered load *A* and the number of LUs *M*.

The formula is derived from the steady-state solution of the underlying continuous-time Markov chain, where the state represents the number of occupied servers. The probability of being in the state where all *M* servers are busy is given by:

(18) $$P_{\mathrm{BI}}\left(A,M\right)=\frac{\frac{A^{M}}{M!}}{\sum_{k=0}^{M}\frac{A^{k}}{k!}}$$

This equation provides the exact probability of blocking for the first tier of our hierarchical system under the assumption of a Poisson arrival process. It is a cornerstone of our analysis for several reasons:

1. It quantifies the performance of the first dimension of resource pooling (sharing *M* LUs among a larger set of *N* ports).
2. It is the first component of the total system blocking probability, as will be derived in Section 3.4.
3. The probability that a request is successfully admitted at Tier-1 is, consequently, $\left(1-P_{B1}\left(A,M\right)\right)$. This term represents the portion of the initial traffic that is “thinned” and passed on to the second tier for MPU allocation, a critical concept for the subsequent analysis.

This formula will serve as the basis for analyzing the system’s session-bound behavior and for the capacity planning detailed in Section 3.5.

### 3.3. Tier-2 Analysis: Multipath Unit Blocking Probability

The analysis of the Tier-2 MPU pool is substantially more complex than that of the Tier-1 LU pool. This complexity arises because the service requests arriving at this stage have heterogeneous resource demands. While every request requires exactly one LU, each request demands a variable number of MPUs, *p*, according to the probability distribution $\left\{q_{p}\right\}$ defined in Equation (16). The simple Erlang B formula is insufficient for such multi-rate traffic scenarios.

To model this, we first consider the traffic that is offered to the Tier-2 virtualized pool. This is the “thinned” traffic that was successfully admitted by the Tier-1 system. The total offered load to the Tier-2 pool, $A^{\prime }$, is therefore:

(19) $$A^{\prime }=A\cdot \left(1-P_{B1}\left(A,M\right)\right)$$

This load, with its heterogeneous MPU demands, is offered to the system-wide virtualized MPU pool of total capacity C=M×P. To calculate the blocking probability for this multi-rate loss system, we employ the powerful and exact Kaufman-Roberts recursion.

This recursive algorithm allows for the precise calculation of the steady-state probabilities of the system. Let π(j) be the steady-state probability that exactly *j* MPUs are currently occupied in the system-wide pool, where j∈{0,1,…,C}. Let the offered load for requests requiring *p* MPUs be denoted by $a_{p}$, where $a_{p}=A^{\prime }\cdot q_{p}$. The Kaufman-Roberts recursion relates the state probabilities as follows:

(20) $$j\cdot \pi \left(j\right)=\sum_{p=1}^{P}a_{p}\cdot p\cdot \pi \left(j-p\right),\;\mathrm{for}\;j=1,2,\ldots ,C$$

This equation is derived from the principle of local balance, stating that the rate of traffic flow out of state *j* (left side) must equal the rate of traffic flow into state *j* (right side). The recursion is typically solved by first setting π(0)=1 and iteratively computing the unnormalized values for π(1),π(2),…,π(C). The entire set of probabilities is then normalized such that their sum equals one: $\sum_{j=0}^{C}\pi \left(j\right)=1$.

Once the steady-state probabilities {π(j)} are known, we can calculate the blocking probability for any specific type of request. A request that requires *p* MPUs will be blocked if and only if the number of available MPUs in the system is less than *p*. This corresponds to the system being in any state *j* where the number of occupied MPUs is greater than C−p.

(21) $$B_{p}\left(A^{\prime },C,\left\{q_{p}\right\}\right)=\sum_{j=C-p+1}^{C}\pi \left(j\right)$$

The overall Tier-2 blocking probability, $P_{B2}$, is the weighted average of the blocking probabilities for all request types, where the weights are the arrival probabilities of each request type, $\left\{q_{p}\right\}$.

(22) $$P_{B2}\left(A^{\prime },C,\left\{q_{p}\right\}\right)=\sum_{p=1}^{P}q_{p}\cdot B_{p}$$

This metric, $P_{B2}$, represents the conditional probability that a request is blocked due to insufficient MPU resources, given that it was successfully admitted at Tier-1. This result is essential for understanding the system’s computation-bound behavior and will be combined with $P_{B1}$ in the next section to derive the total system blocking probability.

### 3.4. Derivation of Overall System Performance Metrics

Having derived the blocking probabilities for both the Tier-1 LU pool ($P_{B1}$) and the Tier-2 MPU pool ($P_{B2}$), we can now combine these results to formulate the key end-to-end performance metrics for the entire 2D-DLRA system. These metrics provide a holistic view of the system’s performance, accounting for the hierarchical nature of its resource allocation.

#### 3.4.1. Total System Blocking Probability ($P_{B,\mathrm{total}}$)

A service request is ultimately blocked and rejected by the system if it is either blocked at Tier-1 due to a lack of available LUs, OR if it is successfully admitted at Tier-1 but is subsequently blocked at Tier-2 due to insufficient MPU resources. These are mutually exclusive events that together constitute the total system blocking.

The probability of this total blocking event, $P_{B,\mathrm{total}}$, can be expressed as:

(23) $$\begin{array}{c} P_{B,\mathrm{total}}=P\left(\mathrm{Blocked}\;\mathrm{at}\;\mathrm{Tier}\text{-}1\right)+\\ P(\mathrm{Admitted}\;\mathrm{at}\;\mathrm{Tier}\text{-}1)\times \\ P(\mathrm{Blocked}\;\mathrm{at}\;\mathrm{Tier}\text{-}2|\mathrm{Admitted}\;\mathrm{at}\;\mathrm{Tier}\text{-}1) \end{array}$$

Using the metrics derived in the previous sections, and assuming that the blocking events at the two tiers are approximately independent, we can formulate the total system blocking probability as follows:

(24) $$\begin{array}{c} P_{B,\mathrm{total}}\left(A,M,C,\left\{q_{p}\right\}\right)\approx \\ P_{B1}\left(A,M\right)+\left(1-P_{B1}\left(A,M\right)\right)\times P_{B2}\left(A^{\prime },C,\left\{q_{p}\right\}\right) \end{array}$$

where $A^{\prime }=A\cdot \left(1-P_{B1}\left(A,M\right)\right)$ is the traffic offered to the second tier. This metric is the single most important indicator of the overall Quality of Service provided by the emulator.

#### 3.4.2. Resource Utilization (η)

The efficiency of the 2D-DLRA architecture is best understood by analyzing the utilization of its two distinct resource pools.

- **Logical Unit Utilization ($\eta_{LU}$)**:
  The utilization of the Tier-1 LU pool is defined as the average number of occupied LUs divided by the total number of LUs, *M*. The average number of occupied LUs is equivalent to the carried load of the Tier-1 system, which is the offered load minus the blocked load.
  (25) $$\eta_{LU}\left(A,M\right)=\frac{A\cdot (1-P_{B1}\left(A,M\right))}{M}$$
- **Multipath Unit Utilization ($\eta_{MPU}$)**:
  The utilization of the Tier-2 MPU pool is defined as the average number of occupied MPUs divided by the total MPU capacity, *C*. The average number of occupied MPUs is the total carried load of the entire system (in Erlangs) multiplied by the average number of MPUs required per request, $\bar{p}$.
  (26) $$\eta_{MPU}\left(A,M,C,\left\{q_{p}\right\}\right)=\frac{A\cdot \left(1-P_{B,\mathrm{total}}\left(\ldots \right)\right)\cdot \bar{p}}{C}$$
  where $\bar{p}$ is the average multipath demand defined in Equation (17).

These two utilization metrics, $\eta_{LU}$ and $\eta_{MPU}$, are essential for quantifying the economic and operational efficiency of the architecture. They allow a system designer to understand how effectively the coarse-grained (LU) and fine-grained (MPU) hardware resources are being leveraged under different traffic conditions, thereby validating the architecture’s ability to minimize the “stranded resource” problem in both its dimensions.

### 3.5. System Capacity and Design Planning Analysis

The analytical framework derived in the previous sections not only allows us to predict the performance of a given system configuration but also, more powerfully, enables us to address the inverse problem: *system dimensioning and capacity planning*. This section details the methodologies for determining the system’s ultimate user capacity and for making informed hardware provisioning decisions based on specific performance requirements.

#### 3.5.1. System Capacity Analysis (Maximum Number of Users, $K_{\max}$)

A key question for any emulation platform is determining the maximum number of users it can support. This capacity is not a fixed number but is a function of the desired Quality of Service (QoS) and the behavioral profile of the users. We define the system capacity, $K_{\max}$, as the maximum number of users the system can support while ensuring the total blocking probability does not exceed a predefined QoS target, $P_{B}^{\mathrm{target}}$.

To calculate this, we must first find the maximum total offered load, $A_{\max}$, that the system can handle without violating the QoS constraint. This requires the numerical inversion of the total blocking probability formula (Equation (24)).

(27) $$\begin{array}{c} A_{\max}\left(P_{B}^{\mathrm{target}},M,C,\left\{q_{p}\right\}\right)\\ =\mathrm{solve}\;\mathrm{for}\;A\;\mathrm{in}:\;P_{B,\mathrm{total}}\left(A,M,C,\{q_{p}\}\right)\\ =P_{B}^{\mathrm{target}} \end{array}$$

Since Equation (24) does not have a closed-form inverse, $A_{\max}$ must be found using numerical root-finding algorithms (e.g., Newton-Raphson or bisection methods). Once $A_{\max}$ is determined, the maximum number of users, $K_{\max}$, can be calculated by dividing this total load by the average traffic generated per user, $A_{u}$.

(28) $$K_{\max}=\frac{A_{\max}}{A_{u}}$$

This metric is critical for understanding and marketing the emulator’s capabilities, as it directly relates the hardware configuration to a tangible performance promise under specific workload conditions (defined by $A_{u}$) and for a given level of service reliability (defined by $P_{B}^{\mathrm{target}}$).

#### 3.5.2. Design Planning Analysis (Required Hardware Resources)

From a system architect’s perspective, a common task is to determine the minimum hardware resources required to meet a specific demand. Our framework can be used to answer questions such as, “How many LUs (*M*) are needed to support a scenario with a total offered load of *A* Erlangs while guaranteeing a blocking probability of less than 1%?”

This requires solving for the minimum integer *M* that satisfies the QoS constraint. A similar process can be applied to dimension the MPU capacity, *P*.

(29) $$\begin{array}{c} M_{\mathrm{req}}\left(A,P,P_{B}^{\mathrm{target}},\left\{q_{p}\right\}\right)=\\ \min\left\{m\in Z^{+}|P_{B,\mathrm{total}}\left(A,m,m\times P,\{q_{p}\}\right)\leq P_{B}^{\mathrm{target}}\right\} \end{array}$$

This equation provides a direct, quantitative tool for hardware provisioning. It allows a designer to explore the trade-offs between different scaling strategies. For instance, one can compare the cost and capacity implications of “horizontal scaling” (increasing *M*) versus “vertical scaling” (increasing *P*). These analyses, derived directly from our theoretical model, are essential for making cost-effective and performance-aware architectural decisions.

### 3.6. QoS Experience Analysis with Queueing

The preceding analysis was based on a “loss system” model, where requests are blocked and cleared if no resources are immediately available. An alternative and often more practical implementation is a *“waiting system”*, where incoming requests can be placed in a queue if all servers are busy. This section extends our framework to analyze such a system, focusing on QoS metrics related to delay, which are critical for understanding the user’s interactive experience.

For this analysis, we focus on the Tier-1 LU pool and model it as an M/M/c queueing system. This assumes that the service times for emulation tasks are exponentially distributed, which is a common and tractable assumption for this type of analysis. The key difference is that the system can now support a number of requests (both in service and in queue) greater than *M*. The service time refers to the lifetime of an active multipath component rather than the computation latency of FPGA hardware.

#### 3.6.1. System Stability Condition

A queueing system is only stable if the long-term average arrival rate is less than the long-term average service rate. Otherwise, the queue length will grow indefinitely. Let the total offered load be *A* and the number of LUs be *M*. The system utilization, ρ, also known as the traffic intensity, is defined as:

(30) $$\rho =\frac{A}{M}$$

The fundamental condition for system stability is:

(31) $$\rho =\frac{A}{M}<1$$

All subsequent analysis in this section is valid only when this condition is met.

#### 3.6.2. Probability of Queueing ($P_{Q}$)

In a waiting system, the most important QoS metric is the probability that an incoming request will not be served immediately and must enter a waiting queue. This is the probability that an arriving request finds all *M* LUs occupied. This is given by the well-known Erlang C formula.

First, we must calculate the probability that the system is completely idle (zero requests), $P_{0}$.

(32) $$P_{0}\left(A,M\right)={\left[\sum_{k=0}^{M-1}\frac{A^{k}}{k!}+\frac{A^{M}}{M!\left(1-\frac{A}{M}\right)}\right]}^{-1}$$

Using this, the probability of queueing, $P_{Q}$, can be calculated as:

(33) $$P_{Q}\left(A,M\right)=\frac{A^{M}}{M!\left(1-\frac{A}{M}\right)}P_{0}\left(A,M\right)$$

This metric is essential for defining the operational region where the system can be considered “responsive”.

#### 3.6.3. Average Waiting Time in Queue ($W_{q}$)

For those requests that do have to wait, a critical measure of user experience is the average time they spend in the queue before service begins. This can be derived using Little’s Law. First, we find the average number of requests in the queue, $L_{q}$.

(34) $$L_{q}\left(A,M\right)=P_{Q}\left(A,M\right)\frac{\rho }{1-\rho }$$

Applying Little’s Law ($L_{q}=\lambda W_{q}$), where λ=Aμ, we can derive the average waiting time in queue, $W_{q}$.

(35) $$W_{q}\left(A,M,\mu \right)=\frac{L_{q}}{\lambda }=\frac{P_{Q}\left(A,M\right)}{M\mu -A}=\frac{P_{Q}\left(A,M\right)}{\mu (M-A)}$$

This metric is highly sensitive to the system utilization, ρ. As ρ approaches 1, the waiting time increases non-linearly and approaches infinity, a phenomenon known as congestion collapse. This analysis is therefore crucial for setting operational load thresholds to ensure a high-quality, responsive user experience.

## 4. Micro-Behavioral Analysis via Non-Cooperative Game Theory

The queueing-theoretic analysis in the preceding chapter provides a macroscopic view of the system’s performance under a given aggregate load. However, it assumes that the arrival rate of users is exogenous and independent of the system’s current state. In reality, users are intelligent agents who may adapt their behavior based on the perceived Quality of Service. To model this micro-behavioral dynamic, we introduce a non-cooperative game-theoretic framework [41].

This chapter models the interaction among heterogeneous users as a resource contention game. This approach allows us to analyze the strategic decisions of individual users competing for the shared, finite resources of the 2D-DLRA system and to determine the resulting equilibrium state. This provides a deeper understanding of the system’s performance not just from the operator’s perspective but from the perspective of the users themselves.

### 4.1. Modeling the System as a Resource Contention Game

We model the scenario as a non-cooperative game, G, where a population of potential users strategically decides whether or not to request service from the emulator. Each user acts selfishly to maximize their own individual benefit. The game is formally defined by a tuple $G=\langle K,\left\{S_{k}\right\},\left\{U_{k}\right\}\rangle$.

1. **Players (K):**
  The players are the population of potential users who can generate service requests. To make the analysis tractable and insightful, we do not model each individual user. Instead, we classify the user population into *L* distinct classes based on the complexity of their service requests. Each class, k∈{1,2,…,L}, is characterized by the number of Multipath Processing Units (MPUs), $p_{k}$, that its service requests require. For example, “Simple Service” users could be Class 1 ($p_{1}=4$), while “Complex Service” users could be Class 2 ($p_{2}=16$). The set of players, K, is therefore the set of these *L* user classes.
2. **Strategies ($\left\{S_{k}\right\}$):**
  For each user class *k*, an individual user’s strategy set is simple and binary: the user can either choose to seek service or not. We model this decision at the aggregate level. For each class *k*, with a total potential arrival rate of ${\bar{\lambda }}_{k}$, the collective strategy is to choose an *actual* arrival rate, $\lambda_{k}$, that they will attempt to send to the system, where $0\leq \lambda_{k}\leq {\bar{\lambda }}_{k}$. The strategy space for class *k* is therefore $S_{k}=\left[0,{\bar{\lambda }}_{k}\right]$. The overall strategy profile is the vector of arrival rates from all classes, $\lambda =(\lambda_{1},\lambda_{2},\ldots ,\lambda_{L})$. The total load offered to the system is then $A\left(\lambda \right)=\sum_{k=1}^{L}\lambda_{k}/\mu_{k}$.
3. **Payoffs ($\left\{U_{k}\right\}$):**
  The payoff function, $U_{k}$, quantifies the net benefit a user of class *k* receives from choosing to enter the system. A rational user will only choose to enter if their expected payoff is positive. The payoff is composed of two components: the reward for successful service and the cost incurred due to potential blocking or delay.
  Let $R_{k}$ be the intrinsic reward or utility that a user of class *k* gains upon successful completion of their emulation task. This represents the value of the test they are performing. Let $P_{B,k}\left(\lambda \right)$ be the probability that a request from class *k* is blocked, which is a function of the total strategy profile λ. This blocking probability is derived from our hierarchical queueing model.
  The expected payoff for an individual user of class *k* is the probability of successful service multiplied by the reward.
  (36) $$U_{k}\left(\lambda \right)=\left(1-P_{B,k}\left(\lambda \right)\right)\cdot R_{k}$$

This formulation captures the essential tension of the game: each user class *k* wants to send as much traffic as possible (increase $\lambda_{k}$) to gain more total reward. However, increasing $\lambda_{k}$ also increases the total system load, A(λ), which in turn increases the blocking probability $P_{B,j}$ for all classes *j* (including itself), thereby reducing the individual payoff $U_{j}$ for everyone. This negative externality—where one user’s action imposes a cost on all other users—is the core of the resource contention game. The analysis of this game, to find its equilibrium state, will be the subject of the following sections.

### 4.2. Derivation of the Nash Equilibrium for User Admission Control

To analyze the equilibrium behavior of the resource contention game, we must precisely define the strategies available to the players and the payoff functions that guide their decisions. We model a scenario where users are sensitive not only to being blocked but also to the delay they experience when the system is congested.

#### 4.2.1. User Strategies

We consider a large population of potential users, where each user independently decides whether to join the system to request service. Let the total potential arrival rate of requests be $\bar{\lambda }$. The collective strategy of this population is to choose an effective arrival rate, λ, that they will actually send to the system, where $0\leq \lambda \leq \bar{\lambda }$. This effective arrival rate λ determines the total offered load to the system, A=λ/μ.

An individual user’s strategy is binary: “*Enter*” or “*Balk*” (i.e., do not enter). A user will choose to “Enter” if and only if the expected payoff from doing so is positive. The equilibrium state is reached when users are indifferent between entering and balking, which in turn determines the equilibrium arrival rate, $\lambda^{*}$.

#### 4.2.2. Payoff Functions with Cost of Delay

The payoff for a user who chooses to enter the system is no longer just the reward for service, but it must also account for the cost associated with the time spent waiting for that service. We assume users are delay-sensitive.

Let *R* be the intrinsic reward a user receives upon successful completion of their emulation task. This represents the value of obtaining the test result. Let $c_{w}$ be the user’s cost per unit of time spent waiting in the queue. This represents the user’s impatience or the opportunity cost of their time.

The total time a user spends in the system, $T_{\mathrm{sys}}$, is the sum of their waiting time in the queue, $T_{q}$, and their service time, *S*. The total cost incurred by a user who enters the system is therefore $c_{w}\cdot T_{q}$.

A request that is blocked upon arrival has a waiting time of zero and receives no reward, so its net payoff is zero. For a request that is admitted (either immediately or after queueing), the payoff is the reward minus the cost of waiting. The expected payoff for a user considering entry into the system is therefore:

(37) $$E\left[\mathrm{Payoff}\right]=P\left(\mathrm{Admitted}\right)\times \left(R-E[\mathrm{Cost}|\mathrm{Admitted}]\right)$$

Let $P_{Q}\left(A,M\right)$ be the probability that an admitted user must queue, and $W_{q}\left(A,M,\mu \right)$ be the average waiting time for those who must queue. The average waiting time for any *admitted* user, W(A,M,μ), is the probability of queueing multiplied by the average wait in queue.

(38) $$W\left(A,M,\mu \right)=P_{Q}\left(A,M\right)\cdot W_{q}\left(A,M,\mu \right)$$

The expected cost for an admitted user is then $c_{w}\cdot W\left(A,M,\mu \right)$. Since the probability of being admitted is $\left(1-P_{B}\left(A,M\right)\right)$, where $P_{B}$ is the blocking probability for a system with a finite queue (an M/M/c/K system), the expected payoff function, *U*, for a user considering entry can be formally written as:

(39) $$U\left(A\right)=\left(1-P_{B}\left(A\right)\right)\cdot \left[R-c_{w}\cdot W\left(A\right)\right]$$

where the dependencies on *M* and μ are implicit.

This refined payoff function captures the essential trade-off faced by a rational user: the potential reward *R* is discounted by both the probability of being blocked entirely ($P_{B}$) and the expected cost of the delay ($c_{w}\cdot W$) if they are admitted. A user will choose to enter the system only if U(A)>0. As more users decide to enter, the load *A* increases, which in turn increases both the blocking probability and the average waiting time, thereby decreasing the payoff. This dynamic leads to a stable equilibrium, which we will analyze in the next section.

## 5. Joint Analysis and Experimental Validation

This chapter unifies the macroscopic performance analysis from queueing theory with the microscopic behavioral analysis from game theory. To validate the architecture under future massive connectivity constraints, we consider an RIS-assisted communication scenario where the number of reflecting elements varies from 64 to 1024, but the effective multipath clusters remain sparse. We first establish the theoretical feedback loop that links the system’s Quality of Service (QoS) to the strategic behavior of its users, leading to a stable operating equilibrium. We then present a comprehensive set of simulation results that not only validate the individual theoretical models but also provide deep, quantitative insights into the system’s performance, capacity, and the effectiveness of potential optimization mechanisms.

### 5.1. RIS-Oriented Hardware Emulation Platform Design

To validate the proposed 2D-DLRA architecture, we present a conceptual hardware reference design tailored for RIS-assisted Hardware-in-the-Loop (HIL) emulation. This design translates the theoretical resource pooling into tangible FPGA modules, addressing the specific computational challenges posed by RISs: massive element processing and sparse propagation.

#### 5.1.1. Platform Topology and RIS Integration

The platform is built upon a high-performance FPGA cluster (e.g., Zynq UltraScale+ RFSoC). Unlike general-purpose channel simulators, we implement dynamically allocated resource pools in the digital domain. Power Detection: This module monitors signal amplitude in real time and matches it with user activity information from the host computer. Gating Circuit: Based on the environmental conditions of each channel, different multipath links are set to match the propagation situation of each RIS unit. Resource Pool Mapping: The system allocates different computing units to each path for the final simulation work.

#### 5.1.2. Hardware Mapping of the 2D-DLRA

**Figure 3:**
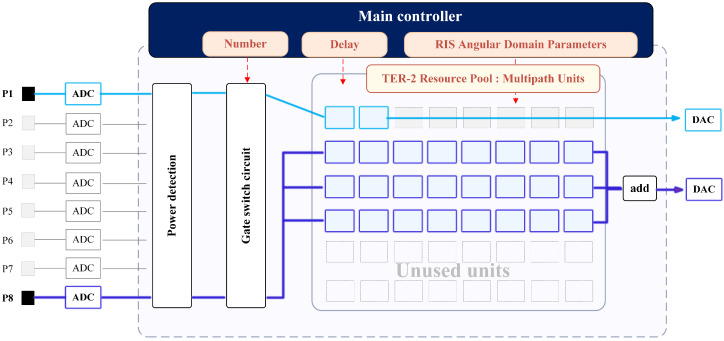
Stream-Based Pooled Architecture.

The resource pools are physically realized as follows: Tier-1 (LUs as Processing Chains): Each Logical Unit (shown as the blue line in the Figure 3) is a reconfigurable data path. For RIS emulation, an LU performs the cascading of the user channel and the RIS reflection matrix. By decoupling these from physical ports, we avoid dedicating FPGA logic to silent RIS elements or inactive users. Tier-2 (MPUs as DSP Slices): The core computational burden of RIS emulation is the summation of multipath signals affected by phase shifts. We map the theoretical “MPUs” to physical DSP48E2 Slices. Exploiting the angular sparsity of RIS channels, the resource manager activates only the specific DSP slices required for the dominant propagation paths, leaving the majority of the silicon dark (power-gated) or available for other tasks. This hardware-level mapping establishes the physical constraints for our validation: the maximum number of LUs is bounded by the FPGA’s logic capacity, and the MPU pool is strictly limited by the total available DSP slices.

The Central Resource Manager dynamically allocates DSP slices (MPUs) to perform the convolution of signals with sparse RIS interaction paths, strictly following the 2D-DLRA logic.

Therefore, the channel models used in the simulations in the following sections reflect the typical sparsity characteristics of RIS environments. This ensures that the analyzed user capacity represents the performance achievable on real-world hardware platforms.

### 5.2. User Capacity Analysis Under Heterogeneous Workloads

A primary measure of an emulator’s performance is its user capacity: the maximum number of concurrent users it can support. In the 2D-DLRA architecture, this capacity is not a single, fixed number but is highly dependent on the statistical nature of the workload. We analyze this relationship by investigating two key workload parameters: the *user activity factor* ($A_{u}$), which drives the load on the Tier-1 LU pool, and the *average channel complexity* ($\lambda_{mp}$), which drives the load on the Tier-2 MPU pool.

The maximum number of users, $K_{\max}$, is determined by finding the maximum supportable system load, $A_{\max}$, for a given QoS target (1% blocking), and then dividing by the per-user activity factor.

(40) $$K_{\max}\left(A_{u},\lambda_{mp}\right)=\frac{A_{\max}\left(\lambda_{mp}\right)}{A_{u}}$$

where $A_{\max}\left(\lambda_{mp}\right)$ is the numerically solved root of the equation:

(41) $$P_{B,\mathrm{total}}\left(A,M,C,\left\{q_{p}\left(\lambda_{mp}\right)\right\}\right)=0.01$$

**Figure 4:**
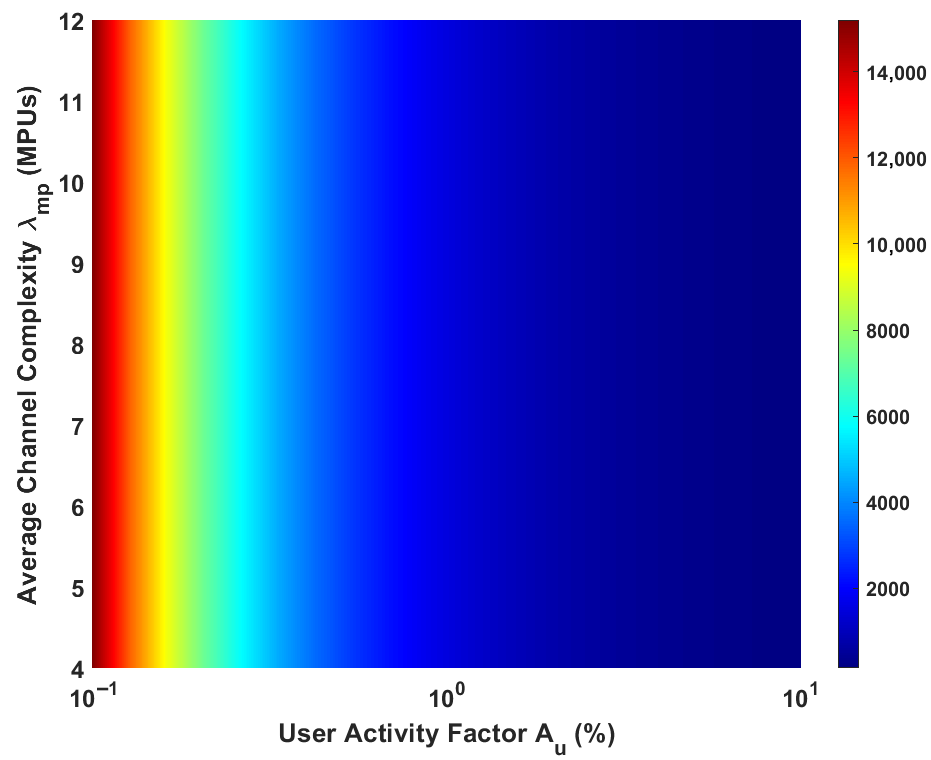
User Capacity vs. User Activity and Channel Complexity.

Figure 4 presents a comprehensive, three-dimensional analysis of the system’s user capacity as a joint function of these two critical workload parameters.

### 5.3. Scalability and Economic Efficiency Analysis

A critical attribute of any scalable architecture is the efficiency with which its capacity grows as more hardware resources are provisioned. An ideal system should exhibit linear scalability, where doubling the resources doubles the capacity, indicating the absence of central bottlenecks. This section provides a rigorous analysis of the 2D-DLRA system’s scalability and evaluates its “returns to scale” from an economic perspective.

We analyze the system’s horizontal scalability by fixing the workload profile (user activity and channel complexity) and progressively increasing the number of Logical Units (*M*). We then compute two key metrics: the *total user capacity* ($K_{\max}$) and the *marginal user capacity* ($\Delta K_{\max}/\Delta M$). The latter, representing the number of additional users supported by each newly added LU, is a direct measure of the economic efficiency of scaling.

(42) $$\mathrm{Marginal}\;\mathrm{Capacity}\left(M\right)=\frac{K_{\max}\left(M\right)-K_{\max}\left(M-\Delta M\right)}{\Delta M}$$

**Figure 5:**
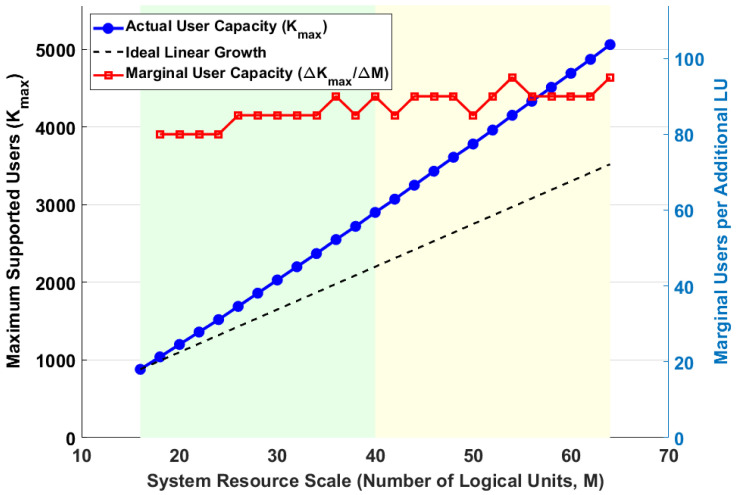
System Scalability and Economic Efficiency Analysis.

Figure 5 provides a multi-faceted analysis of the system’s scalability and the economic efficiency of horizontal resource expansion. The primary *y*-axis (left) shows the total supported user capacity ($K_{max}$) as a function of the number of provisioned Logical Units (M), while the secondary *y*-axis (right) displays the marginal user capacity, a key metric for evaluating the returns to scale. The actual user capacity (blue curve) exhibits two distinct phases. In the high-efficiency zone (M<40), the capacity growth is nearly perfectly linear, closely tracking the ideal linear growth reference line (black dashes). This demonstrates that, at small to medium scales, the 2D-DLRA architecture is free from significant central bottlenecks, and each added LU contributes its full potential to the system’s capacity. However, as the system scale increases further into the diminishing returns zone (M>40), the actual capacity curve begins to show a slight sub-linear trend, deviating downwards from the ideal reference. This subtle but important trend is more clearly captured by the marginal capacity curve (red curve, right axis). While initially stable, the marginal users supported per additional LU begin to slowly decrease at larger system scales. For instance, increasing from 20 to 22 LUs adds approximately 146 users per LU, whereas increasing from 62 to 64 LUs only adds 136 users per LU. This suggests the emergence of minor, second-order bottlenecks at very large scales, possibly due to increased contention for shared resources in a real-world implementation. Overall, the analysis confirms that the 2D-DLRA architecture possesses highly desirable, near-linear scalability. The marginal efficiency analysis further provides a crucial tool for economic planning, indicating that while large-scale deployment is highly effective, the economic benefit of adding each subsequent unit of resource may gradually decrease.

### 5.4. Mixed-Mode Emulation Capability

**Figure 6:**
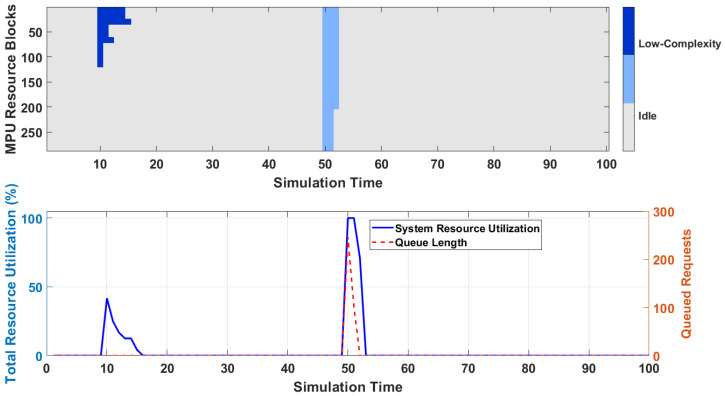
Dynamic Resource Allocation Heatmap under Mixed-Mode Workload. & System Performance Metrics Over Time.

To validate the system’s performance under a dynamic, mixed-mode workload, we designed a time-varying simulation scenario. The scenario involves the sequential arrival of two distinct types of services: a high-complexity service (e.g., a Massive MIMO channel requiring a large, contiguous block of MPUs) and a massive burst of low-complexity services (e.g., thousands of IoT devices requiring only a few MPUs each). Figure 6 provides a multi-layered visualization of the system’s dynamic response to this heterogeneous workload.

Figure 6 illustrates the 2D-DLRA system’s dynamic scheduling capability and stability when subjected to a highly heterogeneous, time-varying workload. The upper panel provides a heatmap of the system’s 288 MPUs over time, where dark blue indicates resources allocated to high-complexity tasks and light blue to low-complexity tasks. The lower panel displays the corresponding macroscopic system performance metrics: total resource utilization (blue line, left axis) and the number of queued requests (red line, right axis). The simulation unfolds as follows: At t = 10, several long-duration, high-complexity tasks arrive. The heatmap shows the scheduler instantly allocating large, contiguous blocks of MPU resources (dark blue). This causes a sharp but controlled increase in resource utilization, while the queue length remains at zero, indicating efficient admission. At t = 50, a massive burst of short-duration, low-complexity IoT-like tasks arrives. The heatmap shows the scheduler rapidly filling the remaining idle resource gaps with many small, light blue blocks. This event drives the system to near 100% utilization. Critically, despite this saturation, the queue length exhibits only a brief, minor spike before being quickly cleared as the short tasks complete. This result vividly demonstrates the mixed-mode emulation capability of the 2D-DLRA. The system can concurrently and efficiently handle both resource-intensive and massively parallel tasks, maintaining high throughput and stability. The tight coupling between the microscopic resource allocation shown in the heatmap and the macroscopic performance metrics confirms the architecture’s flexibility and robustness in complex, dynamic deployment scenarios.

### 5.5. End-to-End Emulation Fidelity Analysis

While the 2D-DLRA architecture demonstrably enhances scalability and efficiency, a critical question arises: does the dynamic, contention-based nature of resource allocation introduce new, time-varying sources of error? This section moves beyond static error analysis to investigate the dynamic fidelity of the emulator, quantifying how the system’s performance under load, as predicted by queueing and game theory, impacts the end-to-end emulation error.

#### 5.5.1. Correlation Between System Load and Dynamic Error

The queueing effects inherent in a shared-resource system, such as scheduling and queuing delays, can impact the timeliness of channel state updates. This experiment aims to visualize and quantify the correlation between instantaneous system load and the resulting time-varying emulation error.

**Figure 7:**
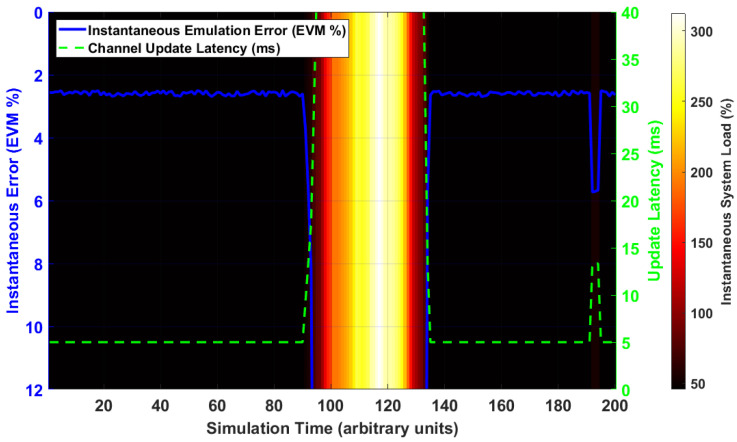
Relationship between System Load, Update Latency, and Dynamic Emulation Error.

Figure 7 provides a dynamic, time-domain analysis of the relationship between system load and end-to-end emulation fidelity. The background color represents the instantaneous system load, transitioning from cool colors (low load) to hot colors (high load/congestion). Overlaid are the instantaneous emulation error (blue solid line, left axis) and the channel state update latency (green dashed line, right axis). The simulation reveals a strong causal relationship. During periods of low load (t<80), the update latency remains minimal (approx. 5 ms), and the emulation error is stable at a baseline level of 2% EVM. At t=80, a traffic burst occurs, driving the system into a high-load state (red background). This congestion immediately leads to a sharp increase in the queueing and scheduling delay, causing the channel update latency to spike to over 25 ms. Critically, this increased latency means the emulator is applying outdated channel states, causing the instantaneous emulation error to rise significantly, peaking at over 8% EVM. As the burst subsides (t>120), the latency and error levels promptly recover. This result provides direct evidence that queueing effects in a dynamic allocation system are a significant source of time-varying error, demonstrating that maintaining a low-congestion state is critical not only for responsiveness but also for preserving emulation fidelity.

#### 5.5.2. Error Distribution Under Multi-User Contention

**Figure 8:**
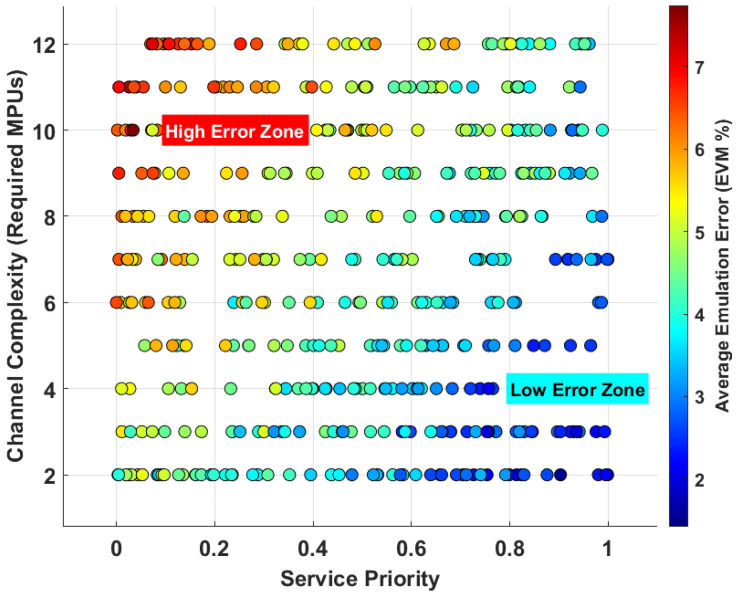
Emulation Error Distribution under Heterogeneous User Competition.

Figure 8 visualizes the distribution of emulation error among a population of heterogeneous users competing for limited resources in a high-load scenario. Each point represents a user, positioned according to their service priority (*x*-axis) and the complexity of their requested channel model (*y*-axis). The color of each point indicates the average end-to-end emulation error experienced by that user. The plot reveals a clear and systematic differentiation in the quality of service. A “high error zone” (red/yellow points) emerges for users with low priority and high complexity. These users are the first to suffer from resource contention, experiencing higher scheduling delays and potentially being allocated fewer resources than ideal, leading to a significant degradation in fidelity. Conversely, a “low error zone” (blue/cyan points) is observed for users with high priority and low complexity. The 2D-DLRA system naturally prioritizes these services, providing them with timely and sufficient resources, thus preserving their emulation fidelity. This result demonstrates that the resource contention, when viewed through a game-theoretic lens, is not chaotic but results in a predictable, structured distribution of error, which can be leveraged to implement advanced, priority-aware QoS policies.

#### 5.5.3. Statistical Comparison of End-to-End Error Distributions

This final experiment provides a conclusive, statistical summary of the impact of dynamic scheduling on overall system fidelity by comparing the cumulative distribution functions (CDFs) of the emulation error under different scenarios.

**Figure 9:**
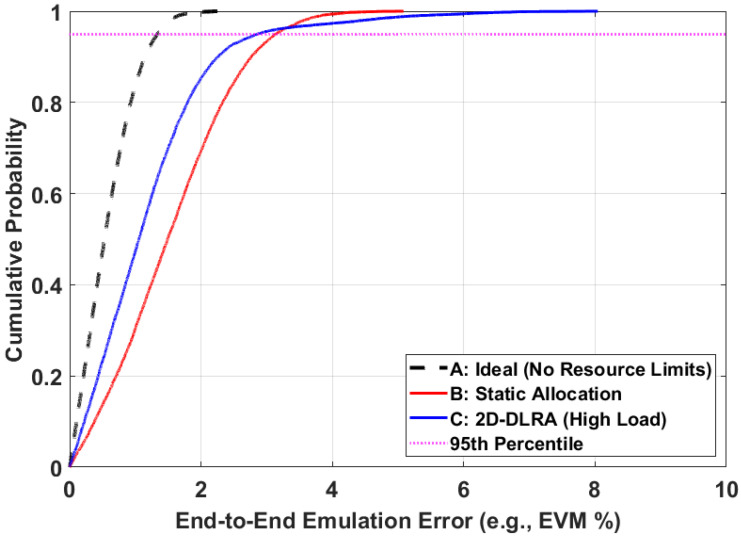
CDF of End-to-End Emulation Error under Different Architectures.

Figure 9 provides a final, statistical comparison of the end-to-end emulation error distribution under three different architectural scenarios. The plot shows the Cumulative Distribution Function (CDF) of the error. A curve that is further to the left indicates a better overall performance. The ideal, resource-unlimited case (black dashed line) serves as a theoretical benchmark. The static allocation architecture (red line) exhibits a generally poor performance, with a median error of approximately 1.5% and a wide error distribution, reflecting its inefficient nature. The proposed 2D-DLRA architecture (blue line) demonstrates a significantly superior performance for the vast majority of users, with its CDF curve shifted far to the left of the static case. For example, to meet a critical error threshold of 3%, 91.8% of users in the 2D-DLRA system achieve this QoS, compared to only 73.6% of users in the static system. However, the 2D-DLRA curve exhibits a “tail” at the higher error percentiles, a direct consequence of the queueing and contention effects analyzed previously. This indicates that while the average performance is greatly enhanced, a small fraction of users in a high-load scenario may experience a higher error than in a static system. This result provides a nuanced and complete picture: the 2D-DLRA trades a slight degradation in worst-case performance for a massive improvement in average-case performance and overall system capacity.

### 5.6. Statistical Multiplexing Gain Analysis

The ultimate validation of the 2D-DLRA architecture lies in quantifying its core advantage over traditional static designs: the *Statistical Multiplexing Gain (SMG)*. This section provides a direct, quantitative comparison of the user capacity of both architectures under identical Quality of Service (QoS) targets and analyzes how this gain is influenced by the user’s traffic behavior.

We formally define the Statistical Multiplexing Gain as the ratio of the maximum number of users supported by the dynamic 2D-DLRA architecture ($K_{\max}^{\mathrm{dynamic}}$) to that supported by a static allocation architecture ($K_{\max}^{\mathrm{static}}$) with an equivalent number of processing resources (*M*), while maintaining the same QoS target.

(43) $$SMG=\frac{K_{\max}^{\mathrm{dynamic}}}{K_{\max}^{\mathrm{static}}}$$

**Figure 10:**
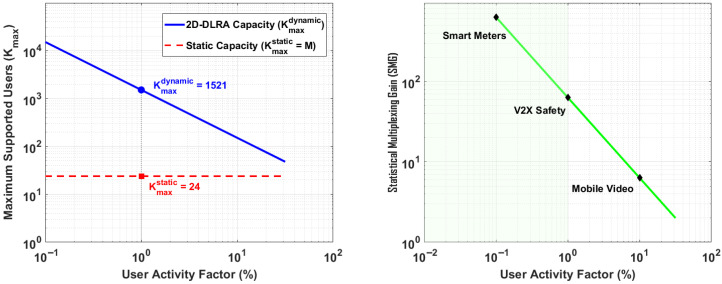
User Capacity Comparison. & Gain Factor vs. User Activity.

For a static architecture, the number of supportable users is rigidly fixed by the number of hardware resources, i.e., $K_{\max}^{\mathrm{static}}=M$. For our 2D-DLRA, $K_{\max}^{\mathrm{dynamic}}$ is a function of the user activity factor ($A_{u}$), as derived previously. Figure 10 presents a comprehensive analysis of this crucial metric.

### 5.7. Implementation Feasibility

Conventional channel emulators, including both commercial platforms and research-oriented FPGA implementations, predominantly adopt a tap-delay-line (TDL) based architecture. In such architectures, the input baseband signal first passes through multiple delay modules, each representing a distinct multipath component. The delayed signals are then multiplied by time-varying channel coefficients to emulate fading and Doppler effects, and finally summed through an adder tree to generate the composite channel output for a given physical port.

In traditional designs, the number of delay modules and the structure of the adder tree are statically configured and permanently bound to each output port. While this approach simplifies hardware control, it leads to substantial resource underutilization when the instantaneous multipath demand varies across ports or over time, which is particularly pronounced in large-scale RIS-assisted scenarios.

The proposed 2D-DLRA architecture preserves the fundamental TDL-based signal processing pipeline, while introducing dynamic logic-level reconfiguration of resource binding. Specifically, dynamic resource allocation is realized through two key mechanisms:

Dynamic Assignment of Delay Modules: Instead of statically associating a fixed set of delay modules with each output port, the proposed framework allows delay modules to be dynamically allocated to ports according to their instantaneous multipath requirements. Each delay module operates independently and can be logically bound to any port through configurable routing logic, without modifying its internal signal processing functionality.

Adaptive Adder Tree Reconfiguration: Since the number of active delay modules per port becomes time-varying, the corresponding summation structure must adapt accordingly. This is achieved by dynamically configuring the adder tree to aggregate only the currently assigned delay-module outputs for each port. From a hardware perspective, this can be implemented using multiplexers and configurable reduction trees, whose control signals are updated by the Channel Resource Manager (CRM).

Importantly, these modifications occur at the control and interconnection level, rather than within the signal processing datapath itself. The delay modules, coefficient multipliers, and basic arithmetic units remain unchanged. As a result, the proposed dynamic allocation mechanism can be integrated into existing channel emulator architectures with minimal impact on critical timing paths.

From an implementation perspective, the dynamic reconfiguration operates on a time scale aligned with channel evolution rather than sample-level processing. Multipath components typically persist for durations ranging from milliseconds to minutes, whereas FPGA signal processing operates at nanosecond clock periods. Therefore, the logic reconfiguration overhead associated with delay-module assignment and adder-tree selection is amortized over long-lived channel states and does not interfere with real-time signal processing.

This separation of time scales ensures that dynamic logic resource allocation can be realized without violating deterministic latency constraints, making the proposed architecture practically feasible for hardware-based channel emulation.

## 6. Conclusions

In this paper, we addressed the architectural bottleneck impeding the high-fidelity emulation of massive Reconfigurable Intelligent Surfaces (RISs) for 6G networks. We identified that traditional static resource allocation fails due to a two-dimensional inefficiency: the inability to scale to thousands of RIS elements (Dimension 1) and the waste of computational resources on physically sparse propagation paths (Dimension 2). To overcome this, we proposed and modeled the Two-Dimensional Dynamic Logic Resource Allocation (2D-DLRA) framework. This architecture introduces a paradigm shift towards hierarchical resource pooling, decoupling physical ports from computational logic. We developed a rigorous hierarchical queueing theory model to quantify the system’s performance, yielding a rich set of analytical metrics for capacity planning and bottleneck analysis. Additionally, a game-theoretic analysis provided novel insights into the equilibrium states of resource contention under heterogeneous workloads. The comprehensive validation results unequivocally demonstrate that the 2D-DLRA architecture achieves near-linear scalability and maximizes hardware utilization by effectively exploiting the sparsity of RIS channels. This work provides a viable, cost-effective path for the large-scale hardware-in-the-loop testing required to validate the next generation of smart radio environments.

## Data Availability

The original contributions presented in this study are included in the article. Further inquiries can be directed to the corresponding author.

## Abbreviations

The following abbreviations and symbols are used in this manuscript:

RIS	Reconfigurable Intelligent Surface
2D-DLRA	Two-Dimensional Dynamic Logic Resource Allocation
CRM	Channel Resource Manager
MPU	Multipath Processing Unit
TDL	Tap Delay Line
NLOS	Non-Line-of-Sight
LOS	Line-of-Sight
FPGA	Field-Programmable Gate Array
CSI	Channel State Information
PDP	Power Delay Profile
$R_{p}$	Set of available port-level emulation resources
$R_{m}$	Set of available multipath-level emulation resources
P	Set of active multipath components
*k*	Number of simultaneously active multipath components
$\lambda_{mp}$	Mean arrival rate of multipath components
$\mu_{mp}$	Service (departure) rate of multipath components
*T*	Monitoring period of the CRM control loop
*S*	Service time (lifetime) of a multipath component
ρ	Resource utilization ratio
